# PRAMEY enhances sperm-egg binding and modulates epigenetic dynamics in bovine embryogenesis

**DOI:** 10.1007/s00441-025-03975-1

**Published:** 2025-05-14

**Authors:** Chandlar Kern, Wan-Sheng Liu

**Affiliations:** Department of Animal Science, Center for Reproductive Biology and Health (CRBH), College of Agricultural Sciences, The Pennsylvania State University, University Park, PA 16802 USA

**Keywords:** PRAMEY, Sperm-egg binding, DNA methylation, Histone methylation, Cattle

## Abstract

Infertility and subfertility are significant reproductive challenges in cattle, often linked to genetic factors. Among these genetic factors, the bovine Y-linked gene family, PRAMEY, has emerged as a candidate due to its involvement in germ cell formation, fertilization, and embryonic development. This study investigates PRAMEY’s role in sperm-egg binding, acrosome integrity, and epigenetic modifications during fertilization and early embryogenesis. Using IVF with bovine spermatozoa treated with either PRAMEY antibody (ab) or rabbit IgG control, we assessed sperm-egg binding and acrosome integrity at 2, 4, and 6 h post-fertilization (hpf). PRAMEY ab treatment doubled sperm binding per oocyte across all time points, with a significant increase at 6 hpf (*P* ≤ 0.05), although no differences in acrosome integrity were observed (*P* > 0.05). To explore PRAMEY’s role in epigenetic regulation, we analyzed DNA (5-methylcytosine (5-mC)) and histone (H3K9me3 and H3K27me3) methylation in zygotes and embryos using immunofluorescent staining techniques. Zygotes derived from PRAMEY ab-treated spermatozoa showed significantly reduced DNA methylation in paternal pronuclei at 10 hpf and maternal pronuclei at 25 hpf (*P* ≤ 0.01). Histone methylation analysis revealed no significant differences in H3K9me3 methylation between groups, but H3K27me3 methylation was significantly lower in embryos produced using PRAMEY ab-treated spermatozoa at the 8-cell and morula stages (*P* ≤ 0.05). In summary, PRAMEY inhibition enhances sperm-egg binding and influences DNA and histone methylation dynamics in bovine embryos, underscoring its potential role in fertilization and early embryonic epigenetic regulation.

**Supplementary Information:**

The online version contains supplementary material available at 10.1007/s00441-025-03975-1.

## Introduction

Fertilization is a complex biological process that involves multiple molecular interactions between sperm and egg, ultimately leading to the formation of a new organism. Understanding the mechanisms underlying successful fertilization is crucial for addressing infertility issues in both humans and livestock species (Bianchi et al. 2022). The process of mammalian fertilization begins with sperm-egg binding, which is mediated by specific molecular interactions between proteins on the surface of both gametes (Miller et al. 1992; Shur & Hall 1982). This binding process is species-specific involving multiple proteins and glycoproteins that ensure successful fertilization while preventing polyspermy (Dilimulati et al. 2022). Recent advancements in structural biology, particularly AI-driven models like AlphaFold, have provided unprecedented insights into the architecture of sperm-egg binding complexes (Elofsson et al. 2024). Essential sperm factors such as PLCZ1 and IZUMO1 have been identified as key mediators of fertilization, significantly advancing our understanding of sperm-egg interactions (Aydin et al. 2016; Ohto et al. 2016; Satouh 2022; Saunders et al. 2002).

Beyond gamete recognition and fusion, successful fertilization depends on precise epigenetic regulation (Dahl et al. 2016; Guo et al. 2014a,b; Inoue et al. 2017; Wang et al. 2018). Following fertilization, both parental genomes undergo extensive reprogramming, including active and passive DNA demethylation as well as histone modifications that regulate embryonic genome activation (Guo et al. 2014a; SanMiguel & Bartolomei 2018; Smith et al. 2012). The paternal genome is rapidly demethylated through TET3-mediated oxidation, while the maternal genome retains higher methylation levels, protected by factors such as H3K9me2 and PGC7 (STELLA/DPPA3) (Han et al. 2018; Loppin et al. 2005; Nakamura et al. 2007, 2012; Zeng et al. 2019). Additionally, histone modifications, such as H3K4me3 (activation) and H3K9me3/H3K27me3 (repression), regulate transcription during embryonic genome activation (EGA) (Bernstein et al. 2006; Dahl et al. 2016; Di Croce & Helin 2013; Simon & Kingston 2009; Zhang et al. 2016). These coordinated DNA and histone methylation dynamics establish pluripotency and regulate early embryonic development (Zhang et al. 2018).

Beyond delivering the paternal haploid genome, spermatozoa also contribute coding and noncoding RNAs (ncRNAS) that may influence embryonic gene regulation and transgenerational inheritance (Jodar et al. 2013; Krawetz 2005; Peng et al. 2012; Song et al. 2011; Wang et al. 2014; Yan et al. 2008). While their precise roles in epigenetic reprogramming remain unclear, sperm-borne RNAs add another layer of complexity to the regulation of early embryogenesis.

In cattle, male fertility is a key determinant of reproductive efficiency in both dairy and beef industries. The bovine Y-chromosome-linked, PRAMEY, gene has been identified as a key regulator of male fertility. Copy number variation (CNV) of PRAMEY is associated with variations in testis development and semen quality (Chang et al. 2011). PRAMEY proteins are enriched in spermatogonia, spermatocytes, elongating spermatids, and mature sperm, indicating a role in germ cell formation and function (Kern et al. 2022; Liu et al. 2017). All genes in the PRAME family encode for leucine-rich repeat (LRR) proteins, which are nuclear receptor transcriptional regulators and cancer-testis antigens (CTAs). They regulate both cancer and germ cell proliferation and differentiation through the retinoic acid (RA) pathway (Chang et al. 2011; Epping et al. 2005; Yang et al. 2023). Besides its function in germ cell development during spermatogenesis, the bovine PRAMEY also plays a role in sperm capacitation, acrosome reaction, sperm-egg interactions, and early embryonic development (Chang et al. 2011; Kern et al. 2022, 2023; Liu et al. 2017). However, the molecular mechanisms underlying these functions remain unclear and require further investigation.

While previous studies have suggested PRAMEY’s involvement in sperm maturation and function (Kern et al. 2022; Liu et al. 2017), its specific roles in sperm-egg binding and epigenetic regulation remain unexplored. Therefore, this study aimed to investigate the role of PRAMEY in two key reproductive processes: sperm-egg binding and epigenetic regulation during early embryonic development. Using antibody-mediated inhibition of PRAMEY during in vitro fertilization (IVF), we examined its effects on sperm binding efficiency and subsequent epigenetic modifications, including DNA methylation and histone modifications. We found that PRAMEY inhibition enhances sperm-egg binding and modulates both DNA and histone methylation at specific developmental time points, suggesting PRAMEY’s importance during both fertilization and early embryonic development. Understanding these mechanisms could provide valuable insights for improving reproductive efficiency in cattle and potentially other mammals.

## Materials and methods

### Embryo production

In vitro production of bovine embryos was conducted as previously described (Kern et al. 2023) unless otherwise noted. Cumulus oocyte complexes (COCs) were obtained by follicle aspiration from ovaries collected immediately post-slaughter at a local abattoir (Nicholas Meat, LLC, Loganton, PA). COCs were matured in 500-μL oocyte maturation media (OMM) consisting of TCM-199 with Earle’s salts, fetal bovine serum, gentamicin, sodium pyruvate, glutamax, Folltropin, and estradiol overlaid with mineral oil. Maturation occurred for 22 h at 38.5 °C and in a humidified atmosphere containing 5% (v/v) CO_2_.

Cauda epididymal spermatozoa were prepared for fertilization following methods described by Kern et al. (2023). Freshly collected testes (from the same slaughterhouse as ovaries) with attached epididymides were transported to the laboratory in a styrofoam box at room temperature within 2 h. Epididymal spermatozoa were flushed from the cauda epididymides with warm HEPES-TALP (Tríbulo et al. 2019) and washed twice by centrifugation (500 × g for 5 min). Spermatozoa from 3 bulls with ≥ 70% motility were pooled in equal proportions. A 300-μL aliquot of washed spermatozoa was added to 3 mL of IVF-TALP (Tríbulo et al. 2019) and incubated for 20 min at 38.5 °C with 5% CO_2_ to facilitate sperm swim up. The supernatant was transferred to a new tube and washed twice (500 × g for 2 min) with HEPES-TALP. The final sperm concentration was adjusted to 25 × 10^6^ spermatozoa/mL prior to treatment with PRAMEY antibody (ab) (5 μg/mL) or rabbit IgG (5 μg/mL) for 30 min at 38.5 °C in a humidified 5% (v/v) CO_2_ atmosphere. The PRAMEY ab was generated using a synthetic peptide (CAQAGLKPEQA) corresponding to a unique PRAMEY sequence (amino acids 167–176, accession no. GU144302.1). The peptide was conjugated to a carrier protein for immunization in New Zealand White-SPF rabbits, and the antibody was affinity-purified following ELISA-based titer determination by New England Peptide, LLC (Gardner, MA, USA). This PRAMEY-specific custom antibody was characterized by Liu et al. (2017). In a previous IVF study (Kern et al. 2023), no differences were observed in sperm-egg binding between the rabbit IgG and Dulbecco’s phosphate-buffered saline (DPBS) control. Therefore, to minimize redundancy while ensuring antibody specificity, the present study used rabbit IgG as the sole control.

For fertilization, spermatozoa (1 × 10^6^) were added to 500 μL of IVF-TALP with ~ 30 matured oocytes per well and co-incubation occurred for 18–20 h unless otherwise noted. For experiments assessing sperm-egg binding and acrosome integrity (see the “Experimental design” section), mature oocytes were denuded of cumulus cells prior to fertilization by washing in HEPES-TALP with hyaluronidase as described previously (Sutovsky et al. 2004). For experiments examining DNA and histone methylation in early embryonic development, cumulus cells were removed post-fertilization by vortexing in HEPES-TALP for 4 min and washing through three wells of the same media.

Zygotes/embryos were collected at various time points based on the experimental design (see the “Experimental design” section). Embryos that were collected 20 h post-fertilization (hpf) were transferred to SOF-BE2 (Tríbulo et al. 2019) covered with mineral oil and incubated at 38.5 °C in a humidified atmosphere of 5% (v/v) O_2_, 5% (v/v) CO_2_, and 90% N_2_ until the designated collection stages (25 hpf and 2-cell, 4-cell, 8-cell, morula, and blastocyst stages). The collected embryos were either used for the present study or frozen (as described by Denicol 2014) and stored in a − 80 °C freezer for future research. The fertilization rates for PRAMEY ab- and rabbit IgG-treated groups were calculated as the number of 2-cell cleaved embryos divided by the total number of oocytes across all IVF experiments in this study.

### Experimental design

#### Sperm-egg binding and acrosome integrity analysis

To investigate PRAMEY’s involvement in sperm-egg binding and acrosome integrity during fertilization, mature oocytes were denuded of cumulus cells by washing in HEPES-TALP containing hyaluronidase as described previously (Sutovsky et al. 2004). Denuded oocytes and spermatozoa treated with PRAMEY ab or rabbit IgG were incubated in IVF-TALP for 2, 4, or 6 h. Potential zygotes were collected at these time points by washing 3 times in DPBS containing 1% (w/v) polyvinylpyrrolidone (PVP). During the first wash, zygotes were pipetted up and down to remove excess spermatozoa. Collected zygotes were fixed and labeled with *Pisum sativum* agglutinin (FITC-PSA) as described below. This experiment was conducted in four replicates, with an average of 73 potential zygotes per time point analyzed for acrosome integrity and sperm-egg binding using FITC-PSA staining.

#### DNA and histone methylation analysis

To determine if PRAMEY influences early embryonic development through epigenetic changes, DNA methylation and histone (H3K9me3 and H3K27me3) trimethylation were analyzed. Oocytes and spermatozoa treated with PRAMEY ab or rabbit IgG were placed in IVF-TALP. Zygotes and embryos were collected at 10, 13, 16, 20, and 25 hpf, as well as at the 2-cell, 4-cell, 8-cell, morula, and blastocyst stages.

For collection up to and including 20 hpf, zygotes were retrieved directly from the IVF wells, cumulus cells were removed, and zygotes washed three times in DPBS containing 1% (w/v) PVP before fixation. For collection at and beyond 25 hpf, cumulus cells were removed at 18–20 hpf, and embryos were cultured in SOF-BE2 until reaching the desired developmental stage. Once collected, embryos were washed three times in DPBS containing 1% (w/v) PVP and fixed (or snap frozen for future research) as described below.

Bovine IVF was performed with at least three technical replicates for each stage of embryo development. Zygotes and embryos (ranging from 5 to 85 per stage) were analyzed for 5-mC and H3K9me3 and H3K27me3 staining (Online Resource 1).

### Immunofluorescent labeling for *Pisum sativum* agglutinin (FITC-PSA)

Labeling for FITC-PSA, 5-mC, H3K9me3 and H3K27me3 was performed at room temperature (RT) in 500 μL volume using 4-well plates unless otherwise specified. Zygotes were washed in DPBS containing 1% (w/v) PVP and fixed in 4% (w/v) paraformaldehyde in DPBS-PVP for 40 min. Fixed zygotes were washed three times in DPBS-PVP, permeabilized in 0.1% Triton X-100 (diluted in DPBS) for 40 min, and washed three more times in wash buffer (DPBS containing 1 mg/mL BSA, 0.1% (v/v) Tween 20). Zygotes were incubated with 30 μg/mL FITC-PSA in DPBS for 30 min to evaluate acrosomal integrity. After staining, zygotes were washed three times in wash buffer and mounted on slides with coverslips using SlowFade Gold Antifade Mountant with DAPI (Thermo Fisher Scientific, catalog no. S36938). Imaging was performed using an Olympus BX51 fluorescence microscope equipped with DP Controller software (Olympus America Inc., Melville, NY). All images were captured with a × 40 objective, maintaining consistent exposure times across all zygotes analyzed.

### Immunofluorescent labeling for 5-methylcytosine

Zygotes and embryos were fixed and permeabilized as described above. Staining for 5-mC was performed as outlined by Yodrug et al. (2021). Embryos were treated with 4 N HCl at 37 °C for 50 min, followed by neutralization through four washes in wash buffer. Non-specific binding was blocked by incubating embryos in DPBS containing 2% (w/v) BSA for 1 h. Embryos were then incubated overnight at room temperature with 1 μg/mL anti-5-methylcytosine (mouse monoclonal; Sigma Aldrich, cat. no. NA81) diluted in DPBS containing 0.05% (v/v) Tween 20 and 1% PVP. Negative controls were prepared using an irrelevant mouse IgG1 antibody (Sigma-Aldrich). After three washes in wash buffer, embryos were incubated with 1 μg/mL goat anti mouse IgG Dylight 488 secondary antibody (Thermo Fisher, cat. no. 35503) at 37 °C for 1 h. Nuclei were counterstained with 50 mg/mL propidium iodide (PI) in DPBS-PVP for 15 min, followed by three additional washes in wash buffer. Embryos were mounted on slides with coverslips using SlowFade Gold Antifade Mountant with DAPI. Imaging was performed using an Olympus BX51 fluorescence microscope with × 40 or × 100 objectives, ensuring consistent exposure settings across all samples analyzed.

### Immunofluorescent double labeling for histones H3K9me3 and H3K27me3 trimethylation

Zygotes and embryos were fixed, permeabilized, and blocked as described above. Embryos were incubated overnight at 4 °C with 1 μg/mL anti-H3K9me3 (mouse monoclonal; Santa Cruz, cat. no. sc-130356) diluted in DPBS containing 0.05% (v/v) Tween 20 and 1% PVP. After three washes, embryos were incubated with 1 μg/mL goat anti mouse IgG Dylight 488 secondary antibody (Thermo Fisher, cat. no. 35503) for 1 h at 37 °C. Following three additional washes in wash buffer, embryos were incubated for 1 h at RT with 1 μg/mL anti-H3K27me3 (rabbit monoclonal; Cell Signaling, cat. no. 9733) diluted in the same buffer. After three more washes in wash buffer, embryos were incubated with 4 μg/mL donkey anti rabbit IgG Alexa Fluor 555 secondary antibody (Thermo Fisher, cat. no. 31572) for 1 h at RT. Finally, embryos were washed three times in wash buffer, mounted on slides with coverslips using SlowFade Gold Antifade Mountant with DAPI. Negative controls were prepared by replacing H3K9me3 and H3K27me3 antibodies with an irrelevant mouse IgG1 antibody. Zygotes were imaged using an Olympus BX51 fluorescence microscope with DP Controller image software (Olympus America Inc., Melville, NY). Imaging was performed with × 40 or × 100 objectives, maintaining constant exposure settings across all zygotes/embryos analyzed.

### Image analysis

Immunofluorescent intensity was quantified using ImageJ software (version 1.8.0_345, NIH, Washington DC, USA). For 5-mC staining, two color channels were analyzed: green (representing 5-mC) and red (representing nuclei). Individual nuclei were identified and outlined using the freehand tool on ImageJ. Paternal and maternal pronuclei were identified based on morphological characteristics, including size, chromatin organization, and spatial positioning within the zygote, which are commonly used for pronuclear distinction in bovine embryos (Beaujean et al. 2004). The area, integrated density, and mean gray value were measured separately for the green and red channels. Values for all analyzed nuclei within a single embryo were averaged to calculate the overall mean for each measurement per embryo. The corrected total cell fluorescence (CTCF) was determined for 5-mC (green) staining using the following formula: CTCF = integrated density – (area of selected cell × mean fluorescence of background readings). This adjustment corrected the integrated density for cell area and background fluorescence. A similar process was used for histone staining (H3K9me3 and H3K27me3). In this case, H3K9me3 was represented in green, H3K27me3 in red, and nuclei in blue. CTCF was determined for H3K9me3 (green) and H3K27me3 (red) staining.

### Statistical analysis

Sperm-egg binding and acrosome integrity data were analyzed using one-way ANOVA followed by Tukey’s post hoc test. Fertilization and cleavage rates, expressed as proportions, were compared between groups using chi-squared (*χ*^2^) tests. DNA methylation (5-mC) and histone methylation (H3K9me3 and H3K27me3) staining were analyzed using one-way ANOVAs and post hoc Tukey tests to compare differences between control and antibody-treated groups.

Due to the collection of ten distinct developmental stages across multiple technical replicates, not all stages were obtained during each trial. This limitation in the experimental design prevented the use of a general linear model (GLM) to statistically evaluate the interaction between treatment (PRAMEY ab vs. rabbit IgG) and stage of embryo development. Consequently, this study does not claim statistical significance for the treatment effect across developmental stages. All data are shown as least-squares means ± standard error of the means (SEM). Statistical significance was defined as *P* ≤ 0.05. All analyses were performed using MiniTab software (version 17, Mintab Inc., State College, PA, USA).

## Results

### Impact of PRAMEY on sperm-egg binding, acrosome integrity, and fertilization

IVF was conducted using PRAMEY ab or rabbit IgG-treated spermatozoa to evaluate PRAMEY’s role during fertilization. Oocytes were assessed at 2, 4, and 6 h post-fertilization (hpf) to examine sperm-egg binding and acrosome integrity. Four rounds of IVF were performed, with 70–81 oocytes evaluated per group.

At each time point (2, 4, or 6 hpf), the PRAMEY ab treatment group exhibited approximately twice as many spermatozoa bound per oocyte compared to the rabbit IgG groups. A significant increase (*P* ≤ 0.05) in sperm-egg binding was observed in the PRAMEY ab treatment group at 6 hpf compared to rabbit IgG (Table 1). The acrosomal integrity of each spermatozoon bound to oocytes was evaluated using PSA staining (Kern et al. 2023). While the percentage of acrosome-reacted (AR) spermatozoa increased over the 4-h period, no significant differences were found between the PRAMEY ab and rabbit IgG groups at any time point (*P* > 0.05) (Table 1). These data are in line with the previous report (Kern et al. 2023), confirming that PRAMEY plays a role in regulating sperm-egg binding.

**Table 1:** Sperm-egg binding and acrosomal integrity*

Treatment	No. of oocytes	HPF	Sperm number/oocyte	% AR sperm
PRAMEY ab	70	2	5.66 ± 0.85^a^	26 ± 3.26^ab^
Rabbit IgG	72	2	2.25 ± 0.24^a^	17 ± 3.51^a^
PRAMEY ab	76	4	7.26 ± 0.82^ab^	30 ± 3.41^ab^
Rabbit IgG	81	4	3.43 ± 0.36^a^	34 ± 4.04^b^
PRAMEY ab	71	6	25.34 ± 2.51^c^	68 ± 4.31^c^
Rabbit IgG	70	6	12.16 ± 1.5^b^	79 ± 3.77^c^

^*^Data are expressed as least-square means ± SEM. Columns with different superscript letters indicate significant differences (*P* ≤ 0.05)

Embryos derived from PRAMEY ab-treated spermatozoa exhibited significantly higher cleavage rates compared to control (*P* ≤ 0.01; Table 2), confirming and expanding upon previous findings (Kern et al. 2023). The proportion of one-cell embryos was comparable between groups, 45.52% in the PRAMEY ab group and 51.02% in the rabbit IgG group. However, from the 2-cell to blastocyst stage, the PRAMEY ab group consistently exhibited increased cleavage progression. Specifically, 40.85% and 26.18% of embryos reached the 2-cell and 4-cell stages, respectively, in the PRAMEY ab group, compared to 37.95% and 16.67% in the rabbit IgG group, with the difference at the 4-cell stage reaching statistical significance (*P* ≤ 0.01; Table 2). Although the cleavage advantage continued through later developmental stages, no statistically significant differences were observed at the 8-cell, morula, or blastocyst stages (*P* > 0.05) (Table 2). These findings suggest a modest developmental advantage conferred by PRAMEY inhibition and support the utility of epididymal spermatozoa for achieving moderate fertilization success in bovine IVF systems.

**Table 2:** The effect of PRAMEY ab on embryo development during IVF*

Treatment	Developmental stages of embryos						
	1-cell	2-cells	4-cells	8-cells	Morula		Blastocyst^#^
PRAMEY ab	188/413* (45.52)	87/213 (40.85)	50/191^a^ (26.18)	30/165 (18.18)		16/122 (13.11)	17/155 (10.97)
Rabbit IgG	226/443 (51.02)	85/224 (37.95)	30/180^b^ (16.67)	24/179 (13.41)		25/195 (12.82)	11/121 (9.09)

^*^Embryo counts at each developmental stage are shown for PRAMEY ab-treated and rabbit IgG control groups. Values represent the number of embryos reaching each stage out of the total number of oocytes observed

^#^The average number of cells per blastocyst was 88 for PRAMEY ab and 86 for rabbit IgG. Different superscript letters within a column indicate statistically significant differences (*P* < 0.01, *χ*^2^ test)

### DNA and histone methylation dynamics in bovine embryos generated with PRAMEY antibody- and rabbit IgG-treated spermatozoa

#### Effect of PRAMEY inhibition on DNA methylation

**Fig. 1:**
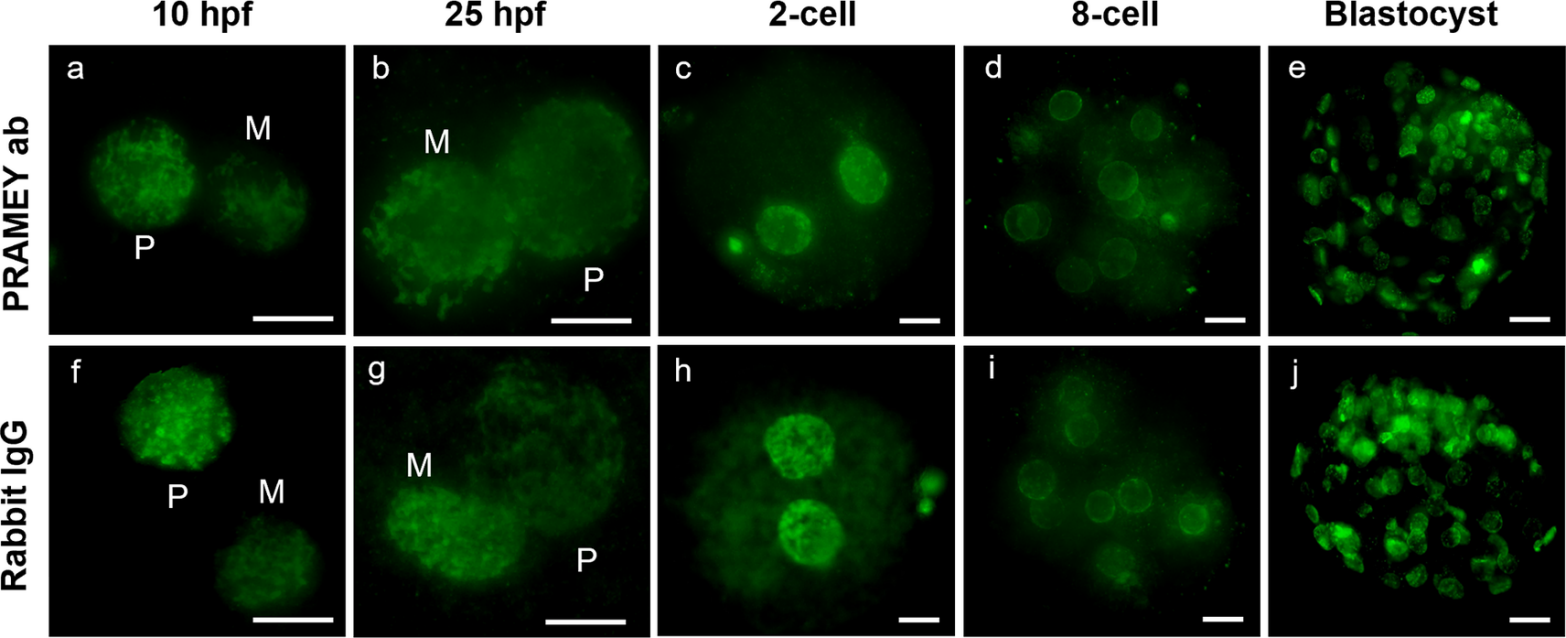
Representative images of embryos produced from PRAMEY ab and rabbit IgG-treated spermatozoa labeled for 5-mC at 10 and 25 hpf and 2-cell, 8-cell, and blastocyst stages. Paternal (P) and maternal (M) pronuclei are marked in 10 and 25 hpf images. Scale bar = 20 µm

To investigate whether PRAMEY influences epigenetic modification during early embryonic development, DNA methylation was analyzed using 5-mC staining across various developmental stages, including zygotes at 10, 13, 16, 20, and 25 hpf and embryos at the 2-cell, 4-cell, 8-cell, morula, and blastocyst stages (Fig. 1). For zygotes (10–25 hpf), 5-mC was measured in both maternal and paternal pronuclei, while for embryos (2-cell to blastocyst stages), 5-mC was quantified in the nuclei of individual cells.

**Fig. 2:**
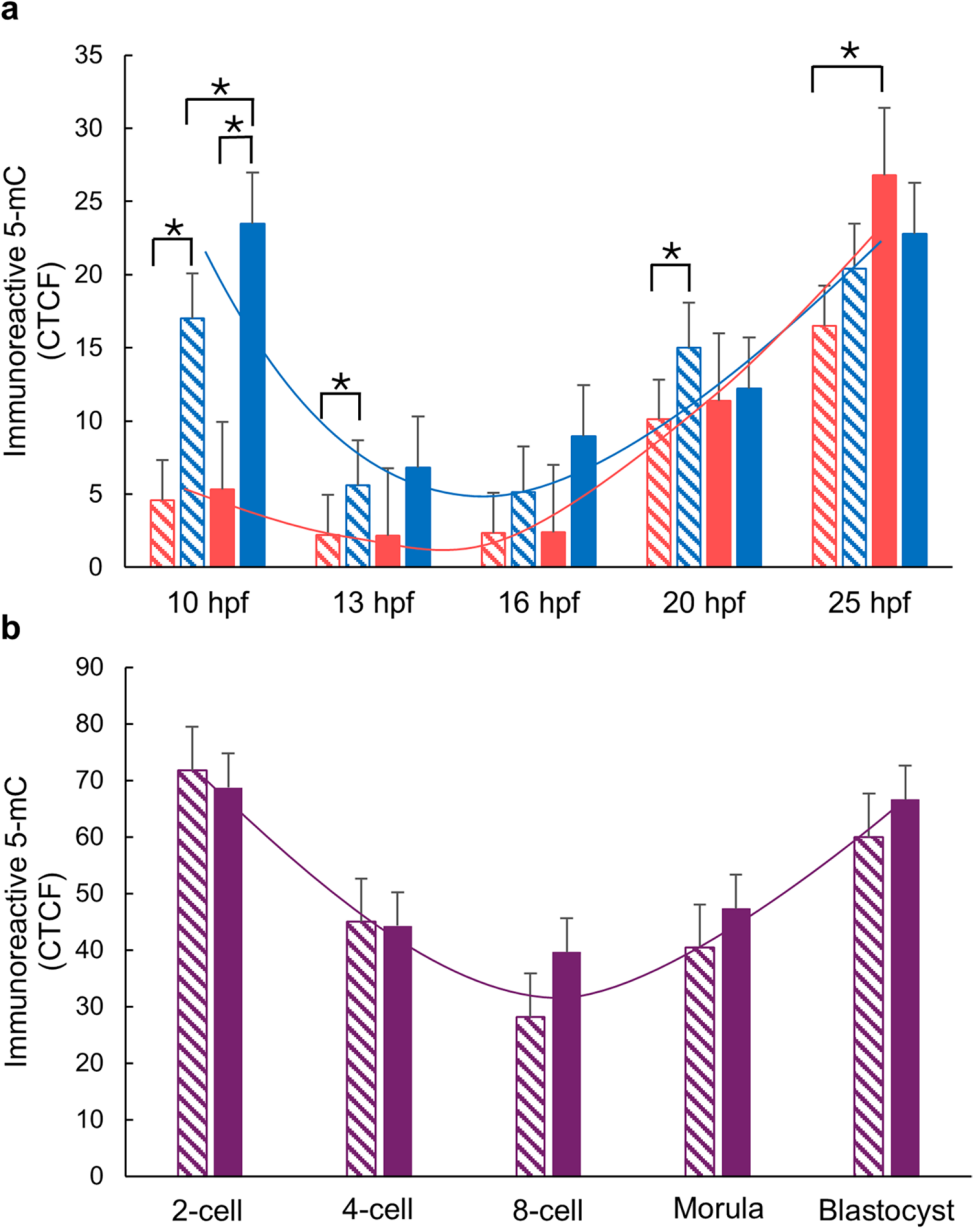
PRAMEY’s effect on 5-mC immunoreactivity during early embryonic development.** a** Paternal (blue) and maternal (pink) pronuclei in zygotes at 10–25 hpf, produced from spermatozoa treated with PRAMEY ab (dashed) or rabbit IgG (solid). **b** Embryos at 2-cell to blastocyst stages (purple). Data represents CTCF for 5-mC. Asterisks (*) indicate significant differences (*P* ≤ 0.05). The blue **a**, pink **a**, and purple **b** lines represent the pattern of methylation in paternal, maternal, and embryonic nuclei, respectively. Data are least-squares means ± SEM of results from 5 to 85 zygotes/embryos per stage

When comparing the PRAMEY ab and rabbit IgG treatment groups, a significant reduction in DNA methylation was observed in paternal pronuclei of the PRAMEY ab group at 10 hpf compared to the rabbit IgG control (*P* ≤ 0.01) (Fig. 2a). Additionally, the maternal pronuclei of the PRAMEY ab group at 25 hpf exhibited significantly lower methylation levels than those in the rabbit IgG group (*P* ≤ 0.01) (Fig. 2a).

No significant differences in DNA methylation were detected between PRAMEY ab and rabbit IgG groups at 13, 16, and 20 hpf, or in embryos at the 2-cell, 4-cell, 8-cell, morula, or blastocyst stages (*P* > 0.05) (Fig. 2a, b). However, distinct patterns of demethylation and remethylation, consistent with previous studies (Dobbs et al. 2013; Park et al. 2007), were observed in both treatment groups: (1) paternal pronuclei underwent demethylation from 10 to 16 hpf, followed by remethylation from 16 to 25 hpf (blue line, Fig. 2a); (2) maternal pronuclei maintained relatively stable methylation levels from 10 to 16 hpf, with remethylation occurring from 16 to 25 hpf (pink line, Fig. 2a); and (3) embryos exhibited a demethylation phase from the 2-cell to the 8-cell stage, followed by remethylation from the 8-cell to the blastocyst stage, forming a characteristic U-shaped pattern (purple line, Fig. 2b). These findings suggest that PRAMEY may influence epigenetic regulation during specific stages of early embryogenesis. Additionally, observation of well-established demethylation and remethylation patterns supports the reliability of our in vitro embryo production system and 5-mC detection methodology.

#### DNA methylation of paternal and maternal pronuclei

We further analyzed DNA methylation in paternal and maternal pronuclei of the 10, 13, 16, 20, and 25 hpf zygotes within each treatment group (PRAMEY ab or rabbit IgG) (Fig. 2a). At 10 hpf, paternal DNA exhibited significantly higher methylation levels compared to maternal DNA in both the PRAMEY ab and rabbit IgG groups (*P* ≤ 0.001) (Fig. 2a). This pattern persisted at 13 and 20 hpf in the PRAMEY ab group, with paternal DNA consistently showing significantly higher methylation than maternal DNA (*P* ≤ 0.05) (Fig. 2a). In contrast, at 13 and 20 hpf in the rabbit IgG group, methylation levels were similar between paternal and maternal pronuclei (Fig. 2a). These findings suggest that PRAMEY inhibition disrupts the typical synchronization of DNA methylation dynamics between paternal and maternal pronuclei, potentially altering the epigenetic reprogramming processes essential for early embryonic development in cattle.

#### Effect of PRAMEY inhibition on histone methylation

**Fig. 3:**
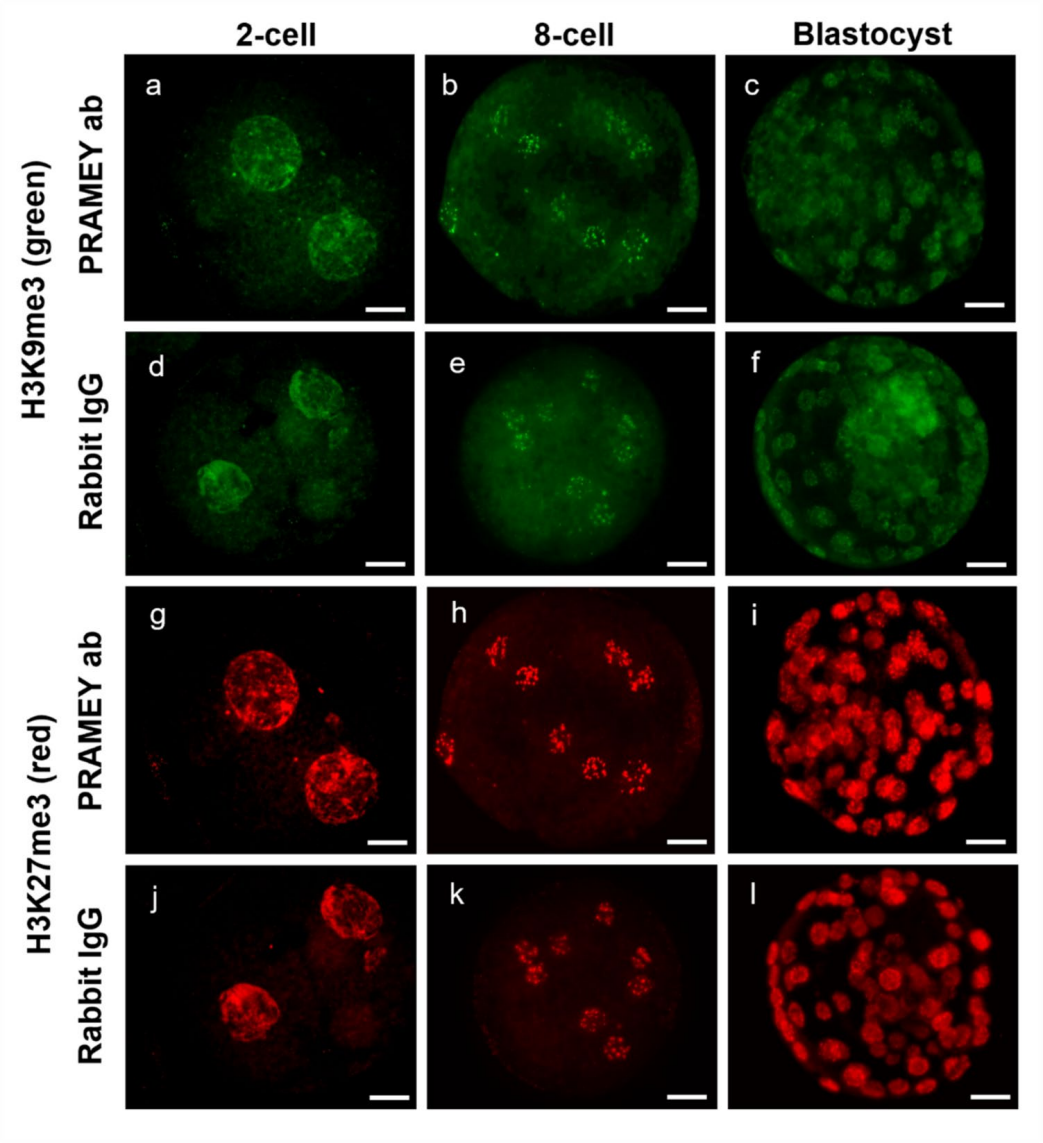
Representative images of embryos produced from PRAMEY ab- and rabbit IgG-treated spermatozoa labeled for H3K9me3 **a**-**f** and H3K27me3 **g**-**l** at 2-cell, 8-cell, and blastocyst stages. Scale bar = 20 µm

To further investigate whether PRAMEY influences epigenetic modification during early embryonic development, histone trimethylation was analyzed using H3K9me3 and H3K27me3 staining across various developmental stages, including embryos at the 2-cell, 4-cell, 8-cell, morula, and blastocyst stages (Fig. 3).

**Fig. 4:**
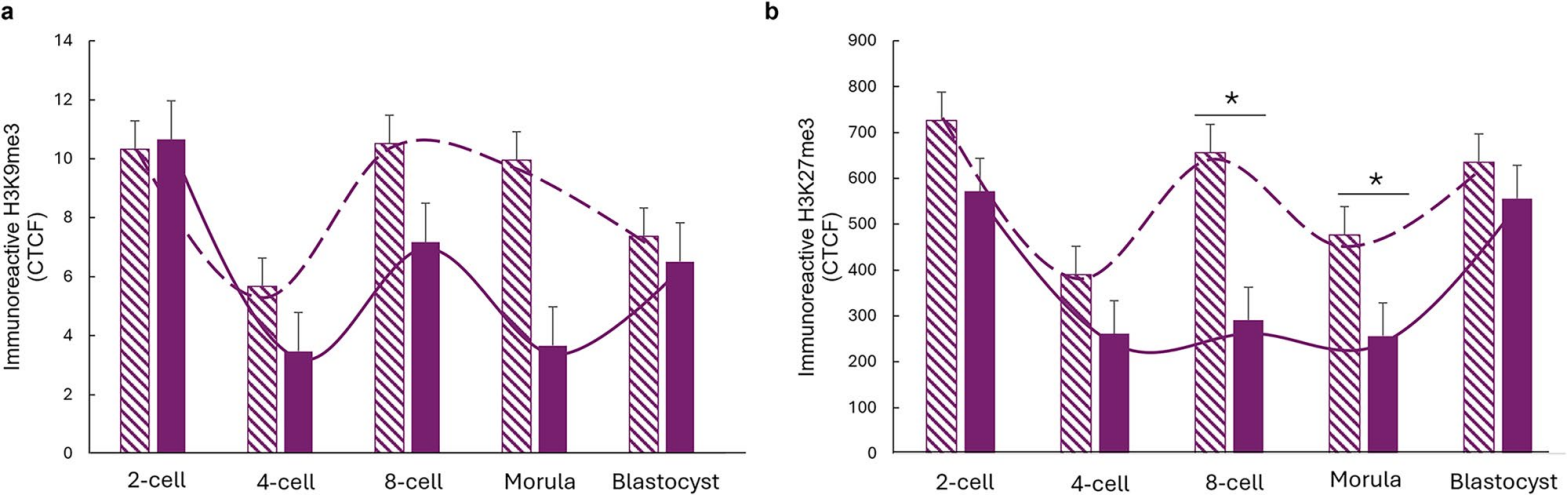
H3K9me3 and H3K27me3 immunoreactivity during embryonic development in embryos produced from PRAMEY ab- (dashed) and rabbit IgG- (solid) treated spermatozoa. **a** H3K9me3 and **b** H3K27me3 immunoreactivity at 2-cell to blastocyst stages. Data represents CTCF for 5-mC. Asterisks (*) indicate significant differences (*P* ≤ 0.05). The purple lines (dashed-PRAMEY ab and solid-rabbit IgG) represent the pattern of methylation in embryonic nuclei. Data are least-squares means ± SEM from 5 to 56 embryos/stage

Histone methylation analysis revealed no significant differences in H3K9me3 methylation levels between the PRAMEY ab and rabbit IgG treatment groups at any developmental stage (2-cell through blastocyst, Fig. 4a, P > 0.05). However, the PRAMEY ab group (dashed line, Fig. 4a) exhibited an earlier onset of histone remethylation from the 4-cell to 8-cell stage compared to the rabbit IgG group (solid line, Fig. 4a), which followed previously reported trends for H3K9me3 (Wang et al. 2018). For H3K27me3 methylation, significant differences between PRAMEY ab and rabbit IgG groups were observed at the 8-cell and morula stages, where the PRAMEY ab group displayed increased methylation levels (Fig. 4b, P ≤ 0.05). The rabbit IgG group (solid line, Fig. 4b) followed a demethylation and remethylation pattern consistent with prior studies on H3K27me3 methylation (Xia et al. 2019). In contrast, the PRAMEY ab group (dashed line, Fig. 4b) displayed a fluctuating pattern, especially between the 4-cell and blastocyst stages, suggesting an alternation in the typical methylation dynamics for H3K27me3. These findings suggest that PRAMEY ab treatment may influence histone methylation patterns, particularly for H3K27me3, during early embryonic development in cattle, potentially altering epigenetic regulation pathways critical for development.

## Discussion

The findings from this study reveal novel insights into the functional role of PRAMEY in bovine fertilization and early embryonic development, highlighting its influence on sperm-egg interactions and epigenetic modifications. PRAMEY inhibition markedly enhanced sperm-egg binding at 6 hpf, consistent with and expanding upon previous observations (Kern et al. 2023). Interestingly, differences at 2 and 4 hpf were not statistically significant, despite numerical increases in the PRAMEY ab-treated group, suggesting that PRAMEY’s regulatory effects may accumulate over time or become more pronounced as fertilization progresses. This time-dependent effect is further supported by the observation that sperm-egg binding increased in both treatment groups between 2 and 6 hpf, likely reflecting ongoing sperm motility and zona pellucida (ZP)/oolemma interactions during prolonged incubation. However, the PRAMEY ab-treated group exhibited significantly higher binding, indicating that PRAMEY inhibition enhances sperm-oocyte interaction beyond what is expected from incubation alone.

Sperm must traverse the cumulus oophorus, ZP, and oolemma to achieve fertilization—barriers that serve as species-specific and polyspermy-preventive checkpoints (Coy & Avilés, 2010; Wassarman & Litscher 2008; Yanagimachi 1994). Previous studies showed that PRAMEY inhibition improves sperm motility (Kern et al. 2023), a trait essential for penetration of these barriers. Thus, enhanced motility may underlie the increased sperm-egg binding observed. Alternatively, PRAMEY may function to limit excessive binding under normal physiological conditions, thereby reducing polyspermy risk. This interpretation is supported by prior findings that PRAMEY inhibition increases the incidence of polyspermy (Kern et al. 2023).

PRAMEY inhibition did not affect acrosomal integrity at 2, 4, or 6 hpf, suggesting that it does not influence acrosomal exocytosis in sperm bound to the oocyte. This contrasts with chemically induced exocytosis studies, where PRAMEY inhibition increased acrosomal integrity (Kern et al. 2023). Differences in acrosome status may be explained by variation in induction method—sperm-egg interaction versus chemical stimulation.

From the 2-cell to blastocyst stage, the PRAMEY ab group consistently exhibited higher cleavage rates compared to the rabbit IgG control. This aligns with previous report indicating that embryos undergoing early cleavage events are more likely to reach the blastocyst stage and possess improved developmental competence (Lenchniak et al. 2008; Magata et al. 2019; Yaacobi-Artzi et al. 2022). Early cleavage is often associated with normal chromosomal content and gene expression patterns conducive to proper embryo development (Fu et al. 2009; Milazzotto et al. 2016). Therefore, the enhanced cleavage rates observed in PRAMEY ab-treated embryos may reflect a broader improvement in developmental potential, underscoring PRAMEY’s role in promoting early embryonic fitness.

Using epididymal spermatozoa allowed for the examination of PRAMEY function without confounding seminal proteins. Although fertilization rates were lower than those typically reported with ejaculated semen (~ 70%) (Sirard 2018; Speckhart et al. 2023), moderate rates observed here confirm the fertilization capacity of epididymal sperm under IVF conditions. The reduced fertilization efficiency may reflect multiple factors. Incomplete sperm maturation, altered membrane composition, and capacitation deficiencies, which are common in epididymal sperm, can impair their ability to interact optimally with the oocyte (Gervasi & Visconti 2017; Nixon et al. 2019; Rickard et al. 2014). Additionally, the absence of seminal-fluid derived tRNA-derived small RNAs (tsRNAs), which have been shown to promote cleavage and developmental competence, may further contribute to reduced fertilization success (Chen et al. 2020).

Importantly, PRAMEY is retained in mature spermatozoa and localizes to the acrosomal and equatorial regions, which are critical for oocyte interaction (Kern et al. 2022, 2023; Liu et al. 2017). Sperm-delivered PRAMEY proteins are released during the acrosome reaction and may reach the oocyte via the sperm tail (Kern et al. 2023). PRAMEY is a Y-linked gene and therefore absent in approximately 50% of embryos. Nevertheless, its paternal contribution may initiate downstream signaling cascades or epigenetic remodeling in the zygote. Although PRAMEY’s presence in mature spermatozoa is well established, we do not necessarily propose that PRAMEY is actively expressed from the early embryonic genome. Instead, we hypothesize that PRAMEY’s role in early embryonic development is mediated through its paternal contribution at fertilization. The observed epigenetic alterations following PRAMEY inhibition suggest that PRAMEY may influence zygote reprogramming and developmental competence through its impact on sperm-egg interactions, epigenetic remodeling, or the transfer of sperm-derived factors (such as RNAs or proteins). These effects could establish a foundation for early transcriptional regulation in the developing embryo, even in the absence of PRAMEY transcription from the embryonic genome.

Consequences of PRAMEY inhibition extend beyond fertilization and impact early epigenetic reprogramming, as demonstrated by significant alterations in DNA methylation dynamics. Specifically, PRAMEY ab treatment resulted in reduced 5-mC levels in the paternal pronuclei at 10 hpf and in the maternal pronuclei at 25 hpf. The reduction in paternal 5-mC aligns with the direct treatment of spermatozoa, but the delayed decrease in maternal methylation suggests more complex mechanisms, potentially involving inter-pronuclear signaling during zygote development. Notably, this accelerated demethylation in zygotes produced with PRAMEY ab-treated spermatozoa may contribute to the increased cleavage rates observed in this and previous studies (Kern et al. 2023).

The maternal genome is traditionally protected from active demethylation by factors such as PGC7 (DPPA3/STELLA) and H3K9me2, which shield maternal 5-mC from TET3-mediated oxidation (Nakamura et al. 2007; Shen et al. 2014). Accordingly, our data reflect a phase of maternal methylation stability from 10 to 16 hpf, followed by a re-establishment of methylation patterns from 16 to 25 hpf, consistent with prior reports (Guo et al. 2014a, b; Inoue et al. 2017). The reduction in maternal 5-mC at 25 hpf in PRAMEY ab-treated zygotes could therefore result from disrupted paternal chromatin signaling, chromatin remodeling asymmetry, or effects mediated through histone modifications. One possibility is that PRAMEY inhibition perturbs histone-mediated crosstalk mechanisms, which in turn influence DNA methylation remodeling in the maternal pronucleus.

Interestingly, we observed that at 10 hpf, 5-mC levels were significantly higher in the paternal pronuclei than in the maternal. This contrasts with prior literature, where a more rapid paternal demethylation has been reported (Park et al. 2007), but may reflect transient developmental asynchrony between the two genomes. Several factors could contribute to this discrepancy, including variable onset of paternal demethylation, heterogeneity in TET3 recruitment, or differences in chromatin accessibility (Canovas et al. 2017; Wrenzycki & Niemann 2003). It is also possible that maternal methylation is not entirely static and begins to undergo passive demethylation earlier than previously appreciated.

Our ability to resolve subtle temporal changes in methylation using precise 5-mC quantification may have uncovered a previously uncharacterized window of transient asymmetry between pronuclei. These findings imply that demethylation of maternal and paternal genomes is not fully synchronized in bovine zygotes. To better characterize these dynamics, future studies employing whole-genome bisulfite sequencing (WGBS) at fine temporal resolution are essential. This ongoing work will provide a comprehensive view of methylation remodeling during the earliest stages of bovine embryogenesis.

PRAMEY also influenced histone methylation, particularly H3K27me3, which was significantly elevated at the 8-cell and morula stages in PRAMEY ab-treated group. H3K27me3 is a key repressive mark associated with transcriptional silencing prior to EGA and plays an essential role in regulating lineage commitment and maintaining pluripotency (Burton & Torres-Padilla 2014; Li et al. 2013; Vastenhouw & Schier 2012; Xu et al. 2021). These findings suggest that PRAMEY influences chromatin states beyond fertilization. This is supported by mechanistic parallels with the PRAME gene family. In somatic systems, PRAME is a known repressor of the retinoic acid (RA) signaling pathway (Chang et al. 2011; Epping et al. 2005; Kern et al. 2021) and regulates the RA-responsive gene *Cdkn1a* (*cyclin-dependent kinase inhibitor 1a*), a critical modulator of cell cycle arrest and embryonic stem cell (ESC) differentiation (Itahana et al. 2016; Liu et al. 1996; Ock et al. 2020). Notably, a mouse Prame family member was shown to repress Cdkn1a via PRC2-mediated H3 K27 me3 enrichment (Napolitano et al. 2020) linking PRAME to polycomb complex-driven transcriptional repression.

Given these precedents, PRAMEY may similarly interface with histone methyltransferase activity to influence *Cdkn1a* expression and modulate developmental timing, cell cycle progression, or pluripotency maintenance during bovine preimplantation development. Elevated H3K27me3 in PRAMEY ab embryos could reflect compensatory chromatin remodeling in response to disrupted PRAMEY signaling, with downstream effects on transcriptional control and lineage decisions. These observations further reinforce PRAMEY’s role as an epigenetic regulator during early embryogenesis.

Overall, these findings support a functional role for PRAMEY during fertilization and early embryonic development. Its effects on sperm-egg binding and early epigenetic reprogramming provide a foundation for understanding sperm-derived regulatory mechanisms in bovine embryos. However, the extent to which PRAMEY’s paternal influence persists into later developmental stages remains to be determined. Future studies should explore whether PRAMEY inhibition impacts lineage specification, EGA, and blastocyst formation to better understand its role in early embryogenesis. Additionally, research should examine how PRAMEY-mediated events at fertilization influence subsequent developmental outcomes using methods such as whole genome bisulfite sequencing (WGBS), ICM/trophectoderm quantification, and lineage tracing (Dobbs et al. 2013; Duan et al. 2019; Zhu et al. 2018).

## Supplementary Information

Below is the link to the electronic supplementary material.

Supplementary file1 (DOCX 23 KB)

## Data Availability

All data generated and analyzed during this study are included in this publication.
