# Supplementary material for: PRAMEY enhances sperm-egg binding and modulates epigenetic dynamics in bovine embryogenesis
Source: Cell Tissue Res. 2025 May 14;401(1):83–94. doi: 10.1007/s00441-025-03975-1 (PMC12222268; doi:10.1007/s00441-025-03975-1)
Supplement: Supplementary file 1 — Supplementary file1 (DOCX 23 KB) [file 441_2025_3975_MOESM1_ESM.docx]

**Online Resource 1**. Number of zygotes/embryos stained for 5-mC, H3K9me3, and H3K27me3*.

| Developmental Stage | No. of Embryos: 5-mC | | | No. of Embryos: H3K9 & H3K27 | | | |
| --- | --- | --- | --- | --- | --- | --- | --- |
|  | PRAMEY ab (n) | Rabbit IgG | PRAMEY ab | | Rabbit IgG | | |
| 10 hpf | 57 | 85 | N/A | | | N/A |  |
| 13 hpf | 7 | 8 | N/A | | | N/A |  |
| 16 hpf | 7 | 8 | N/A | | | N/A |  |
| 20 hpf | 51 | 60 | N/A | | | N/A |  |
| 25 hpf | 66 | 65 | N/A | | | N/A |  |
| 2-cell | 31 | 35 | 56 | | | 50 |  |
| 4-cell | 30 | 12 | 20 | | | 18 |  |
| 8-cell | 13 | 12 | 17 | | | 12 |  |
| Morula | 8 | 15 | 8 | | | 10 |  |
| Blastocyst | 12 | 5 | 5 | | | 6 |  |

*Each developmental stage contains the total sum of zygotes/embryos stained for 5-mC, H3K9me3, and H3K27me3 from 3 or more technical replicates of IVF.
